# Diversity in Translation: Equal Is Greater Than

**DOI:** 10.1111/cts.70699

**Published:** 2026-08-27

**Authors:** Elvin T. Price, John A. Wagner

**Affiliations:** ^1^ VCU Convergence Health Outcomes Virginia Commonwealth University Richmond Virginia USA; ^2^ School of Pharmacy Virginia Commonwealth University Richmond Virginia USA; ^3^ Aditum Bio Cambridge Massachusetts USA; ^4^ Clearsite Bio Bound Brook New Jersey USA; ^5^ Trames Bio Cambridge Massachusetts USA

**Keywords:** diversity, equity, inclusion, translation

“*Of all the forms of inequality, injustice in health care is the most shocking and inhumane*.”*—* *Martin Luther King Jr. (speech to the Medical Committee for Human Rights, 1966)*

Clinical and translational science is designed to reduce uncertainty. We interrogate mechanisms, link biomarkers to clinical endpoints, characterize exposure‐response relationships, and use quantitative methods to measure and predict benefit and risk. When the evidence base is less representative than the intended population, the result is not only inequitable, but also weaker science. Uncertainty may appear for any specific population, including older adults, children, pregnant persons, patients with organ dysfunction, individuals from diverse ancestral or geographic backgrounds, rural communities, transgender patients, or people in environments where access, culture, adherence, and trust determine whether a medicine can fulfill its intent.

The Clinical and Translational Science (CTS) Diversity, Equity, and Inclusion (DEI) special collection was assembled around the simple premise that diversity is a prerequisite for high‐quality translation. Across the articles summarized in Table 1, the collection spans clinical trial representativeness, ethnic sensitivity, pharmacogenomics, pediatrics, pregnancy, biomarker validity, community engagement, workforce education, global development, and decision science. Collectively, the papers argue that inclusion is increasingly a scientific and operational requirement for generalizable evidence.

**TABLE 1:** Brief summary of articles in the CTS DEI Special Collection.

Reference	Title	Primary DEI focus	Contribution
1	How Clinical Pharmacology Can Support Clinical Trial Diversity and Inclusion	Trial diversity and inclusion as a clinical pharmacology deliverable	Articulates CP levers (design, exposure‐response, bridging) that operationalize inclusion
2	Critical Roles for Modeling and Simulation and Real‐World Evidence to Inform Challenges in Clinical Trial Diversity Planning	Quantitative methods enabling diversity	Positions M&S/RWE as tools to plan, monitor, and defend generalizable evidence
3	Pharmacokinetics, pharmacodynamics, and safety of asundexian in healthy Chinese and Japanese volunteers, and comparison with Caucasian data	East Asian populations; bridging	Generates comparative PK/PD evidence supporting globally generalizable dosing
4	Ethnic Sensitivity Assessment of Mosunetuzumab Pharmacokinetics and Pharmacodynamics in Chinese Patients With Relapsed or Refractory Follicular Lymphoma	Chinese patients; oncology bridging	Supports dosing confidence across populations for equitable access to oncology innovation
5	Inter‐Ethnic Differences in the Efficacy and Safety of Tyrosine Kinase Inhibitors Used in Oncology: Insights From Phase 3 Clinical Trials	Cross‑ethnic benefit‑risk differences	Highlights where “one label fits all” may be scientifically weak without subgroup evidence
6	New Horizon in Clinical Development Strategy in Japan Based on New Guideline on Japanese Phase 1 Studies Prior to Multi‐Regional Clinical Trials	Japan; pathway to inclusive MRCTs	Connects regulatory strategy to practical inclusion in global development programs
7	Representation of Older Adult Patients in Clinical Trials	Older adults; trial representativeness	Defines and motivates improved inclusion of older adults to strengthen external validity
8	Age‐related off‐label drug prescribing in pediatric patients in South Korea and consistency of labeling compared to the United States, Europe, and Japan	Pediatrics; labeling equity across regions	Maps pediatric evidence gaps via off‑label use and cross‑region labeling discordance
9	Pharmacogenomic studies of fertility outcomes in pediatric cancer survivors – A systematic review	Pediatric cancer survivorship; fertility; PGx	Synthesizes what is (and isn’t) known on cancer survivorship and fertility
10	Pharmacokinetic model‐guided enoxaparin dosing in the Neonatal ICU: Retrospective cohort study to plan for prospective feasibility trial	Neonates; dosing	Uses model‑guided dosing to reduce uncertainty where evidence is historically sparse
11	Predicting the exposure of mycophenolic acid in children with autoimmune diseases using a limited sampling strategy: A retrospective study	Pediatric autoimmune disease; access‑friendly TDM	Reduces burden of monitoring while improving exposure estimation in children
12	Pharmacodynamics of Aspirin Through Gestation: Predictors of Aspirin Response and Association With Pregnancy Outcome, a Prospective Cohort Study	Pregnancy; physiologic change across gestation	Links PD response variability to outcomes, supporting evidence‑based tailoring in pregnancy
13	Frequency and Implications of High‐Risk Pharmacogenomic Phenotypes Identified in a Diverse Australian Pediatric Oncology Cohort	Diverse pediatric oncology; actionable PGx	Quantifies high‑risk PGx burden to support equitable preemptive testing strategies
14	Large‐Scale Analysis of Age and Sex Effects on Corrected QT and JT Intervals: Insights From Hospital‐Based Controls and Moxifloxacin‐Treated Cases	Age/sex; balanced safety benchmarking	Establishes reference ranges that reduce misclassification across age/sex strata
15	Perspectives on Using Pharmacogenomics to Guide Tobacco Cessation: Survey Results From an American Indian Community	Indigenous community; acceptability/trust in PGx	Brings community voice into PGx translation, which is important for uptake and validity in practice
16	Pharmacogenetics of tamoxifen in breast cancer patients of African descent: Lack of data	African descent; underrepresentation	Flags a concrete precision‑medicine gap with direct implications for treatment equity
17	Inadequate representation of individuals of African ancestry in pharmacogenetics of tamoxifen research	African ancestry; research representativeness	Makes underrepresentation itself the scientific problem—because it undermines generalizability
18	Reply to “Inadequate representation of individuals of African ancestry in pharmacogenetics of tamoxifen research”	Evidence gap clarification	Reinforces or refines the critique—useful for sharpening the field's consensus and next steps
19	The needs and gaps in pharmacogenomics knowledge and education among healthcare professionals in Malaysia: A multisite Delphi study	Workforce education; global implementation	Defines actionable education priorities to enable equitable PGx adoption across settings
20	The impact of skin color and tone on histamine iontophoresis and Doppler flowmetry measurements as a pharmacodynamic biomarker	Skin tone; biomarker validity	Demonstrates how measurement bias can distort inference—driving more equitable biomarker design.
21	The Edges of Clinical Pharmacology: A Narrative Review on Religious and Sociocultural Influences in Drug Management	Religion/culture; adherence and acceptability	Treats sociocultural context as a determinant of exposure, adherence, and outcomes
22	Gender‐Affirming Hormone Therapy: Pharmacokinetic Considerations for Oncology in Transgender Patients	Transgender patients; GAHT–oncology interactions	Frames gender‑affirming care as essential context for safe, inclusive oncology pharmacology
23	Informing Inclusive Engagement in Clinical Research: Insights From a Long‐Standing Community‐Based Wellness and Prescription Produce Program	Community partnerships; sustained engagement	Offers concrete engagement lessons to improve participation and retention (and thus validity)
24	Pharmacokinetics of a Novel Piperaquine Dispersible Granules Formulation Under Fasting and Various Fed Conditions Versus Piperaquine Tablets When Fasted in Healthy Tanzanian Adults: A Randomized, Phase I Study	Endemic‑setting population; formulation usability	Generates PK evidence directly in a relevant population to support globally appropriate use
25	Predictors of potential clinically harmful drug–drug interactions at the medical wards in the University of Benin Teaching Hospital, Benin City	LMIC inpatient setting; safety	Identifies predictors of harm to inform safer prescribing where resources may be constrained
26	A case study of inclusion of rural populations in research: Implications for science and health equity	Rurality; geographic access	Treats “place” as a determinant of who gets studied and how results generalize to practice
27	Bioavailability of three novel oral, sustained‐release pellets, relative to an immediate‐release tablet containing 500 mg flucytosine: A randomized, open‐label, crossover study in healthy volunteers	Usability/adherence via formulation	Formulation strategies can broaden real‑world usability, an often‐overlooked inclusion lever
28	How debunking biases in research and development decisions could lead to more equitable healthcare?	Decision bias; equitable prioritization	Connects cognitive/organizational bias to inequitable evidence generation and how to counter it

Abbreviations: GAHT, gender‐affirming hormone therapy; LMIC, low‐ and middle‐income countries; MRCTs, multi‐regional clinical trials; PGx, pharmacogenomic; TDM, therapeutic drug monitoring.

The title phrase of this editorial, Equal Is Greater Than, is intended to be provocative, but not as a political slogan. Equal, in this editorial, means equal attention to the heterogeneity of bodies, biology, identity, environment, geography, and lived experience that shapes drug exposure, response, safety, adherence, access, and outcomes. When that heterogeneity is treated as a legitimate scientific variable rather than noise, inconvenience, or a late‐stage demographic reconciliation exercise, the resulting science is improved.

## 1 DEI As Translational Methodology

In public discourse, DEI is often described as ideology. In clinical pharmacology and translational science, it is more useful to understand DEI as methodology. The relevant questions are familiar to every translational scientist. Are we measuring variables that plausibly explain variability in exposure, response, safety, adherence, and outcomes? Are trial participants sufficiently representative of the patients who will receive the therapy in practice? When we extrapolate across subgroups, are we doing so with evidence, or are we relying on convenience and assumption? Methodologic assessment includes questions around bias, confounding, missingness, effect modification, model performance, and external validity.

Clinical pharmacology is central to this discussion because it provides the quantitative language for making diversity actionable. PK/PD, exposure‐response analysis, biomarkers, pharmacogenomics, modeling and simulation, and real‐world evidence can convert inclusion goals into dosing, safety, and effectiveness guidance. When historically underrepresented groups are absent from discovery data sets, early trials, pivotal programs, or implementation studies, development programs produce knowledge optimized for the most studied populations. That is the opposite of what translational science is intended to deliver.

## 2 Diversity as a Design Principle

A central theme of this special collection is that diversity can be operationalized just as it is used to advance drug development. Liao et al. describe how clinical pharmacology can support clinical trial diversity and inclusion, including through thoughtful design, dose selection, exposure‐response characterization, and bridging strategies [1]. Kadambi et al. extend that logic by emphasizing the roles of modeling and simulation and real‐world evidence in diversity planning [2]. Together, these papers move inclusion from aspiration to development plan.

The papers focused on ethnic sensitivity and global development make a similar point. Comparative PK/PD and safety studies in Chinese and Japanese volunteers, ethnic sensitivity assessment of mosunetuzumab in Chinese patients with relapsed or refractory follicular lymphoma, inter‐ethnic comparisons of benefit–risk for oncology tyrosine kinase inhibitors, and evolving development strategy in Japan all illustrate the value of deliberately generating evidence that can support or refute extrapolation [3, 4, 5, 6]. The scientific goal is to design programs capable of demonstrating when differences matter, when they do not, and how much uncertainty remains.

A homogeneous clinical development program cannot prove similarity across heterogeneous populations. It can only fail to examine difference. Diversity should therefore not be framed solely as a recruitment challenge. It should be framed as a design constraint. If thought of as a design constraint, it requires the same operational approach required for other design issues, including early planning, sufficient resources, quantitative monitoring, explicit assumptions, and willingness to adjust the program when the evidence is not adequate for the intended use.

## 3 Beyond Recruitment

The collection also makes clear that inclusive translation extends beyond enrollment targets. The problem is not simply who enters a trial, but what variables are measured, how outcomes are interpreted, whose biology is represented in discovery data sets, whether biomarkers perform similarly across populations, whether formulations and monitoring strategies are usable in practice, and whether communities have reason to trust the research enterprise.

Table 1 summarizes the breadth of this perspective. The articles on older adults, pediatrics, neonates, pregnancy, QT assessment, pediatric oncology, and autoimmune disease show that age, development, physiology, and life stage are not peripheral considerations [7, 8, 9, 10, 11, 12, 13, 14]. The pharmacogenomics articles focused on African ancestry, American Indian communities, pediatric oncology, fertility, and Malaysia demonstrate the risk of precision medicine becoming precision inequity when discovery, validation, education, and implementation are unevenly distributed [9, 13, 15, 16, 17, 18, 19]. The biomarker article on skin color and tone shows that measurement itself can encode bias if the technology performs differently across the people in whom it is applied [20]. Articles addressing religion, sociocultural influences, gender‐affirming hormone therapy, rural populations, community engagement, Tanzanian adults, drug–drug interactions in Nigeria, flucytosine formulation, and decision bias extend the frame from biological representation to implementation in real settings [21, 22, 23, 24, 25, 26, 27, 28].

The point is not to fragment translational science into an endless series of subgroups. The point is to make assumptions behind generalization explicit. In some cases, a subgroup analysis, a limited‐sampling strategy, a bridging study, or a model‐based extrapolation argument will be sufficient. In other cases, dedicated enrollment, new measurement methods, local evidence generation, or postmarketing commitments will be needed. What is unacceptable is to leave the uncertainty invisible.

## 4 From Aspiration to Operation

Holistically, the CTS DEI special collection describes a field moving from aspiration to method. Several practical commitments follow (Table 2). Representativeness should be specified prospectively, monitored quantitatively, and resourced as a core development objective. Measurement and reporting should be standardized so evidence can aggregate across studies and settings. Generalizability should be supported by evidence rather than assumed. Participation should be made feasible through lower‐burden designs, model‐informed dosing, community partnerships, and operational choices that reduce avoidable barriers.

**TABLE 2:** Lessons learned from diversity in translation.

#	Principle	Lesson learned	References
1	Treat representativeness as a design constraint	Inclusion goals should be pre‐specified, resourced appropriately, monitored quantitatively, and adjusted when off track, like any other core development goal	[1, 2]
2	Standardize measurement and reporting	If we want evidence to aggregate across studies and settings, we need common data elements and transparent subgroup reporting, with attention to privacy and community governance where appropriate	[15, 19]
3	Quantify generalizability rather than assuming it	When enrollment is limited in key subgroups, development programs should include explicit generalizability arguments, bridging logic, and post‐marketing plans that make uncertainty visible and reducible	[3, 4, 5, 6]
4	Build methods that make participation feasible	Limited‐sampling strategies, model‐based dosing, and logistics that reduce burden enable inclusivity	[10, 11, 23, 26]
5	Prevent precision inequity	Pharmacogenomics and biomarkers require diverse discovery datasets, ancestry‐aware validation, and candid reporting of performance across populations, along with capacity building so implementation does not remain confined to well‐resourced centers	[9, 13, 15, 16, 17, 18, 19]
6	Recognize that trust, culture, and place are translational variables	Community partnership, sociocultural competence, and geographic access constraints shape what translation looks like in practice and therefore belong in study design	[21, 23, 26, 29]

The same operational logic applies to pharmacogenomics and biomarkers. Diverse discovery data, ancestry‐aware validation, transparent performance reporting, and implementation capacity are required if precision medicine is to be precise for more than the best‐resourced populations. Trust, culture, and place should also be treated as translational variables because they shape recruitment, adherence, retention, access, and implementation. None of these ideas is outside clinical pharmacology. They are extensions of the same quantitative and mechanistic thinking that already defines the discipline.

This framing is particularly important at a time when the terminology of DEI is contested. The field and society can debate language, but it cannot debate the reality that biology, environment, context, and access modify exposure, response, safety, and health outcomes. Nor can translational scientists ethically or scientifically ignore the consequences of generating evidence primarily in populations easiest to study.

## 5 Equal Is Greater Than

Equal is greater than because equality, practiced as rigorous inclusion, produces greater validity. It reduces hidden uncertainty where that uncertainty is currently concentrated. It prevents predictable failures of generalization. It improves the ability of model‐informed development, exposure‐response analysis, biomarkers, pharmacogenomics, and real‐world evidence to support dosing, safety, efficacy, and implementation decisions for the patients who will use the medicines being developed or prescribed.

Clinical and translational science holds the promise of moving discoveries from bench to bedside and, increasingly, from bedside to bench to back to bedside. That promise will be incompletely fulfilled if the evidence base does not reflect the people, settings, and clinical circumstances in which therapeutics are used. The CTS DEI special collection should therefore be read not as a separate social agenda, but as a translational science agenda. Diversity is not a distraction from rigor. It is one of the conditions of rigor.

In clinical pharmacology and translational science, equal is greater than when equal means equal scientific attention to the variability that determines whether medicines work, for whom, at what dose, with what risk, and in what setting. That kind of equality yields something greater: better evidence, better decisions, and better health.

## Funding

The authors have nothing to report.

## Conflicts of Interest

J.A.W. is employed by Aditum Bio, Clearsite Bio, and Trames Bio, and may own stock and/or stock options. E.T.P. declares no conflicts.
